# The IgA Vasculitis Study: the evolution of a cross-sectional cohort into a trial-ready cohort

**DOI:** 10.1093/rap/rkaf099

**Published:** 2025-08-21

**Authors:** Chloe E C Williams, Julien Marro, Andrew J Chetwynd, Joseph Brown, Nikolaos Skoutelis, Silothabo Dliso, Catherine McBurney, Louise Oni

**Affiliations:** Department of Nephrology, Royal Liverpool and Broadgreen University Hospital Trusts, Liverpool, UK; Department of Women’s and Children’s Health, Institute of Life Course and Medical Sciences, University of Liverpool, Liverpool, UK; Department of Women’s and Children’s Health, Institute of Life Course and Medical Sciences, University of Liverpool, Liverpool, UK; Centre for Proteome Research, Department of Biochemistry, Cell and Systems Biology, Institute of Systems, Molecular and Integrative Biology, University of Liverpool, Liverpool, UK; Department of Women’s and Children’s Health, Institute of Life Course and Medical Sciences, University of Liverpool, Liverpool, UK; Department of Paediatric Nephrology, Alder Hey Children’s NHS Foundation Trust Hospital, Liverpool, UK; National Institute for Health and Care Research Alder Hey Clinical Research Facility, Clinical Research Division, Alder Hey Children’s NHS Foundation Trust, Liverpool, UK; National Institute for Health and Care Research Alder Hey Clinical Research Facility, Clinical Research Division, Alder Hey Children’s NHS Foundation Trust, Liverpool, UK; Department of Women’s and Children’s Health, Institute of Life Course and Medical Sciences, University of Liverpool, Liverpool, UK; Department of Paediatric Nephrology, Great Ormond Street Hospital for Children, London, UK; Department of Renal Medicine, UCL Centre for Kidney and Bladder Health, Royal Free Hospital, London, UK

**Keywords:** Henoch–Schönlein purpura, paediatric, children, kidney, nephritis

## Abstract

**Objectives:**

The aim of this report is to describe a single-centre cohort study and its evolutionary stages into a trial-ready cohort with the vision of stopping kidney failure secondary to IgA vasculitis (IgAV).

**Methods:**

The IgA Vasculitis Study was established as a single-centre, cross-sectional cohort study recruiting children with a clinical diagnosis of IgAV and has evolved into a trial-ready framework. Sociodemographic and clinical data, as well as corresponding biosamples, were collected longitudinally and the natural history of the first 100 recruits is provided.

**Results:**

The IgA Vasculitis Study commenced in June 2019. The first 100 children recruited to the study had a mean age of 7.3 years (s.d. 3.7) and a male:female ratio of 1.5:1. At presentation, all children had a lower limb–predominant rash, 76% had musculoskeletal involvement, 43% gastrointestinal involvement and 23% met the definition of nephritis. Most children (54%) were discharged after 6 months, however, 17% required paediatric nephrology input. The mean timing of onset for nephritis was 25.5 days (s.d. 22.9) following disease presentation (range 0.0–101 days). Fourteen children with IgAV nephritis (IgAV-N) received immunosuppression. Children with an older age, residing in more affluent areas, with gastrointestinal involvement or a positive urine dipstick (for proteinuria and/or haematuria) at presentation had greater odds of developing nephritis. Children with IgAV-N had statistically significantly more hospital visits and unplanned hospital admissions (*P* < 0.001).

**Conclusion:**

Nephritis remains a serious consequence of IgAV with little evidence to guide management. This report outlines an exemplar study to advance the field towards better interventions.

## Graphical Abstract

**Figure:**
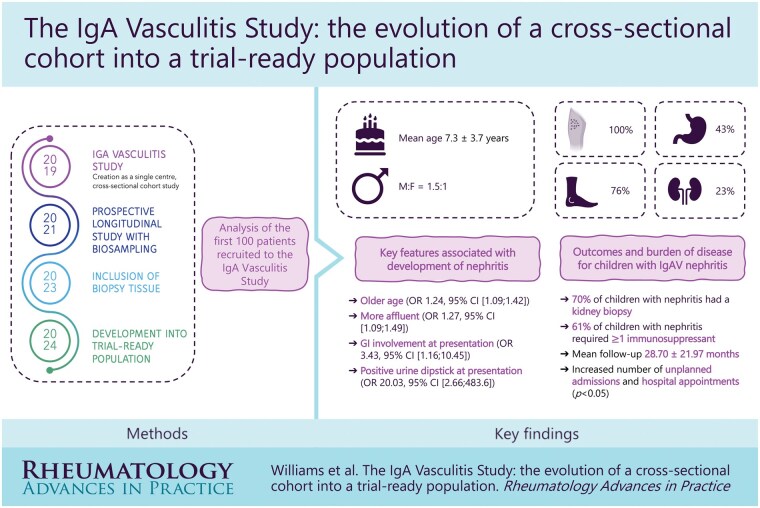

Key messages

- Clinical trials are urgently needed for patients with IgAV at highest risk of developing nephritis.
- Proteinuria at presentation may be a good predictor of nephritis in children with IgAV.
- Children with nephritis or atypical disease courses have a higher burden of disease that should be addressed.

## Introduction

Immunoglobulin A vasculitis [IgAV; formerly known as Henoch-Schönlein purpura (HSP)] is the most common paediatric vasculitis, with an estimated annual incidence of 3–27 cases per 100 000 children [1–3], and it can present at any age. It has a peak age at presentation of 4–6 years, with up to 90% of childhood-onset cases presenting before 10 years of age [4, 5]. Adult-onset disease is ultra rare, with an incidence of ≈0.1–3 per 100 000 adults [6]. IgAV appears to have some geospatial clustering around the Mediterranean and the western continental regions [7], with some ethnic disparities, including a higher incidence in Caucasian and Asian ethnic groups compared with children of Black ethnicity [4, 8].

IgAV typically presents with a characteristic lower limb–predominant non-blanching purpuric rash, less commonly affecting the upper limbs, torso and, rarely, the face [9]. In classification tools, the rash is a mandatory criterion together with one of four other criteria: abdominal pain, histopathological evidence of IgA, arthritis or arthralgia or kidney involvement [10]. IgAV involves the musculoskeletal system in up to 90% of patients, gastrointestinal (GI) system in up to 70%, and/or kidney systems in ≈30% of patients, with heterogeneity in terms of severity within each of the presenting systems [11]. Joint pains and GI symptoms mostly contribute to the significant short-term morbidity, however, they rarely manifest as serious long-term complications [12]. An underrecognized consequence is persisting or recurrent disease, seen in 2.6–66% of children [13]. Around 20–30% of children experience kidney inflammation, termed IgAV nephritis (IgAV-N), and there is a consistently reported risk of 1–2% progressing to irreversible kidney failure [14, 15]. This risk increases significantly in selected cohorts presenting with more severe clinical or histological features of nephritis. In adult-onset IgAV, limited evidence suggests a far greater risk of chronic kidney disease (CKD), with reports of 23% of elderly patients developing kidney failure [5]. Reducing the risk of CKD is therefore a priority in this condition. Following diagnosis, it is recommended that all children complete 6 months of kidney monitoring with periodic urinalysis [16–18]. Advances to risk stratify patients are yet to be made; however, a normal urinalysis at 7 days post-diagnosis has been shown to be an excellent predictor of normal kidney outcomes, implying that stratification may be achievable [19]. Even in children who develop significant proteinuria, many will have a complete recovery, and the outcomes for most children are excellent, with 94% of children disease-free 2-years post-diagnosis [19–21].

Several cohort studies have described the natural history of IgAV-N, including a very large multicentre European retrospective analysis of 1148 children [22]. However, there continue to be barriers in generating high-quality evidence, with the Cochrane Collaboration repeatedly concluding that there remains a lack of high-quality randomized controlled trials to prevent or treat kidney disease in people with IgAV and no evidence to suggest any one immunosuppressive agent is superior when compared with others [23]. There is an urgent need to progress the field to reduce the incidence of kidney failure related to this condition.

The aim of this work is to describe the clinical features, disease course and outcomes of the first 100 children recruited to a study dedicated to IgAV and to describe the evolution of a single-centre cohort study into an age-inclusive, trial-ready framework as an incremental move towards evidence generation.

## Patients and methods

### The IgA Vasculitis Study

The IgA Vasculitis Study was established with two main aims: to advance the discovery of scientific targets for treatment and to understand the natural history of the disease to guide optimal interventional opportunities. It began as a single-centre, prospective, observational cohort study recruiting participants attending a large tertiary children’s hospital: Alder Hey Children’s NHS Foundation Trust, Liverpool, UK. The aim of the study was to collect baseline clinical data with a corresponding biosample resource. Following protocol amendments, it evolved into a longitudinal study to capture the first 12 months of the disease course with additional amendments to extend the bioresource to include access to any surplus biopsy tissue taken for clinical purposes. The study subsequently received funding to extend the resource to permit national recruitment with the lead site supporting remote data collection and biosampling to form a trial-ready population through a ‘consent to recontact’ cohort.

### Patient cohort

Children ≤18 years of age with a clinical diagnosis of IgAV were eligible to be recruited to the IgA Vasculitis Study. For the purposes of this report, the first 100 patients recruited to the longitudinal aspect of the IgA Vasculitis Study are described.

### Data collection

Data were collected prospectively and longitudinally and enriched where necessary. This included comprehensive demographic data at disease onset, as well as symptom onset, date of diagnosis, date of first presentation and possible disease triggers. Indices of multiple deprivation (IMDs) were used to describe socio-economic status. English postcodes were determined using the 2019 version of the postcode lookup tool [24] and a similar tool was used for Welsh postcodes [25]. Each postcode corresponds to a decile, with the first decile being most deprived and the tenth being most affluent. Data were also collected on clinical observations (blood pressure and temperature), laboratory results, urinalysis and treatment. The number of accident and emergency (A&E) centre visits and hospital admissions relating to the diagnosis of IgAV were also recorded. Inpatient hospital admissions solely for the purpose of conducting investigations such as a kidney biopsy were excluded. Data were collected at time intervals aligned with the previously published monitoring schedule, the Alder Hey HSP Pathway [19].

### Definitions

In line with the schedule, patients were discharged if there were no abnormalities on urinalysis 6 months after diagnosis. Patients were considered lost to follow-up if they did not attend three consecutive appointments. The child’s last review was defined as the most recent review prior to discharge or their most recent clinic review as of June 2023. Multisystem involvement at presentation was defined as patients who presented with clinical features in two or more organ systems and children were considered febrile if they had a recorded temperature ≥38°C. A vasculitic rash extending beyond the lower limbs was defined as extensive while a severe rash was defined as a rash that was also accompanied by necrotic, bullous and/or ulcerative lesions. At the time of recruitment to the cohort, there were no agreed, standardized definitions of recurrent or persisting disease, therefore they were defined if they had been coded as such by a paediatric rheumatologist or nephrologist. ‘Atypical disease’ here refers to either recurrent or persisting disease [13, 18]. Urinary abnormalities were defined as a urine dipstick positive for blood, protein or both. IgAV-N was defined as a urine albumin:creatinine ratio (UACR) >30 mg/mmol, aligned with international guidelines [17]. Kidney histology was described using the International Study of Kidney Disease in Children (ISKDC) classification or Oxford MEST-C classification [26]. IgAV-N was assumed absent in children lost to follow-up.

### Statistical analysis

Statistical analyses were performed using GraphPad Prism version 10.1.2 (GraphPad Software, San Diego, CA, USA) and R version 4.3.2 (R Foundation for Statistical Computing, Vienna, Austria). Due to the relatively small cohort, it was assumed that the data were non-parametric. Two-tailed Mann–Whitney U tests were performed on continuous data while two-sided Fisher’s exact tests were performed on non-continuous data. Spatial mapping of deprivation was performed using the tmap package in R [27] and patient postcodes were geocoded using the Postcodesio R package [28]. Exploratory analysis was performed of the baseline characteristics associated with urinary abnormalities at 6 months and only children who completed 6 months of follow-up were included. Odds ratios (ORs) and confidence intervals (CIs) were calculated using Fisher’s exact test for binary categorical variables and univariate logistic regression for other variables. The association of any blood test findings and the clinical outcomes were explored through categorical variables (i.e. normal or abnormal based on the age-specific laboratory reference ranges used in our centre [29]). *P*-values <0.05 were considered statistically significant.

### Ethical and regulatory approval

Patients included in this study were consented to the IgA Vasculitis Study, which had full approval by the Health Research Authority and Health and Care Research Wales (HCRW) (REC 17/NE/0390, protocol UoL001347, IRAS 236,599). Written informed consent and/or assent was obtained from the caregiver and/or patient.

## Results

**Figure 1.**
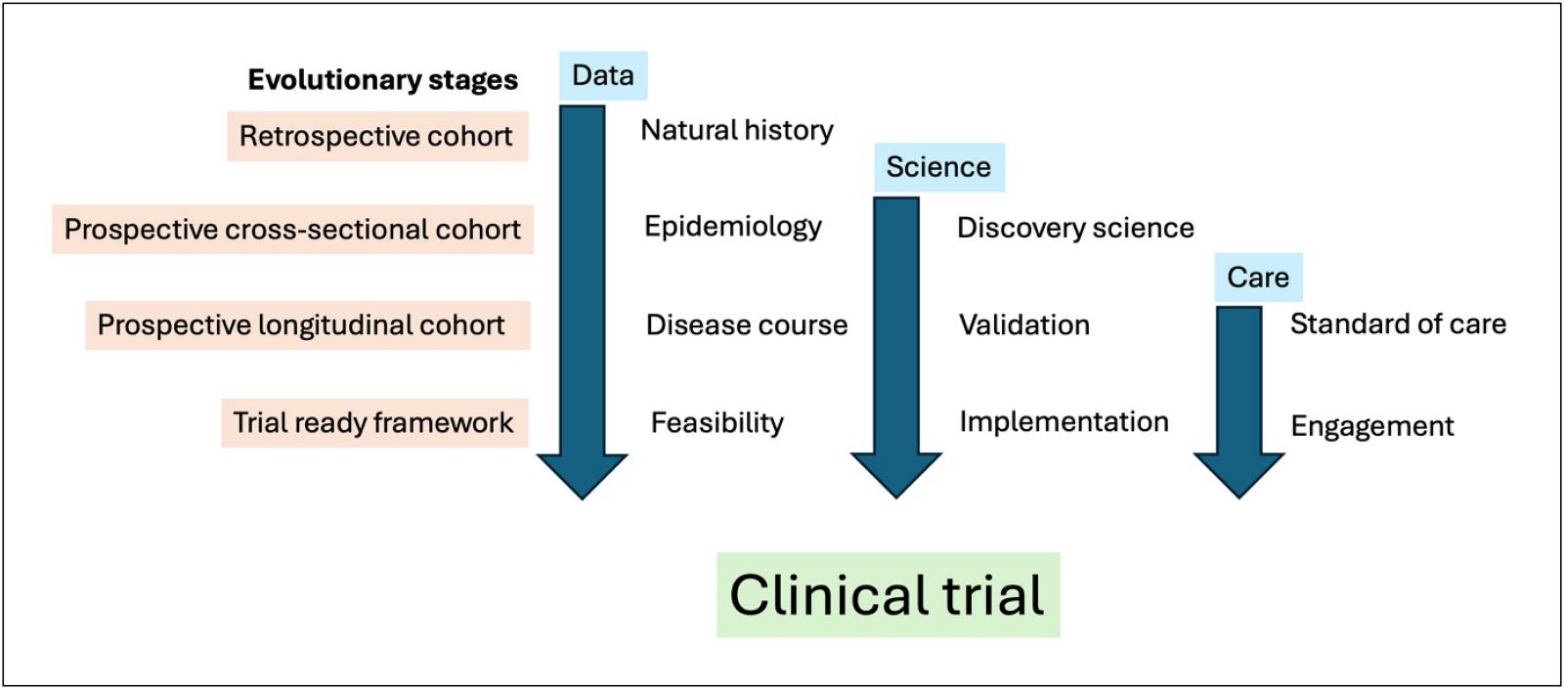
The evolutionary stages of the IgA Vasculitis Study. The IgA Vasculitis Study was established as a cross-sectional study following a previously published retrospective cohort analysis and the data were gathered in June 2023 [19]. The study had an amendment to incorporate longitudinal data and biosampling in January 2021, the use of surplus histology tissue was incorporated in January 2023, the study end date was extended following further funding in August 2023 and in August 2024 there were approval submissions to create a national trial-ready population via a ‘consent to recontact cohort’. To date the cohort has incorporated patient engagement [43], supported the first national standard-of-care guidelines, developed biopsy guidance [44], standardized the definitions of atypical disease, compiled natural history data contained within this manuscript and previous work [19] and contributed to scientific understanding [13, 18]. Further work in progress includes a disease-specific vasculitis activity scoring tool, scientific data on the IgA protein structure and a workshop to create a pathway to design a clinical trial

**Figure 2.**
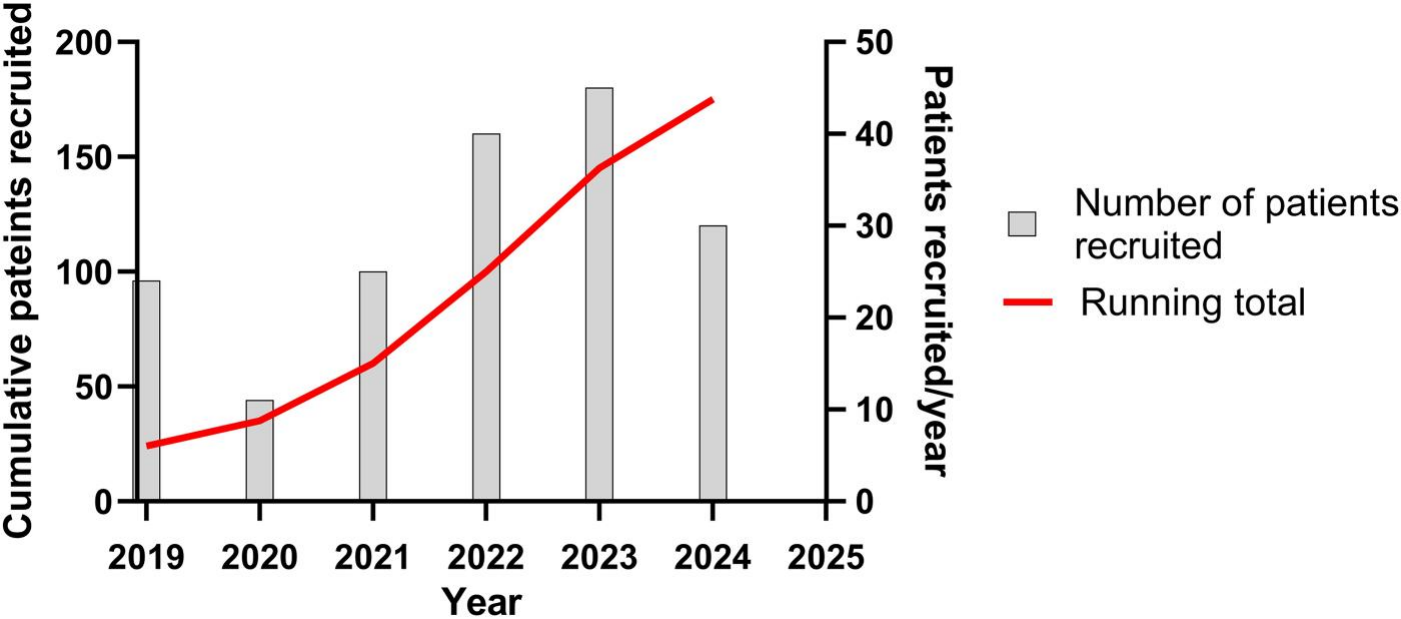
Annual recruitment figures for the IgA Vasculitis Study. As of August 2024, there were 175 patients recruited to the study and over the 5 years since inception there was a mean recruitment rate of 2.9 children per month (annual recruitment range 11–45 patients). There was a notable reduction in recruitment figures during 2020 due a period of study closure during peak outbreaks of the COVID-19 pandemic and therefore recruitment figures for 2022–23 are likely to be the most representative to date.

**Figure 3.**
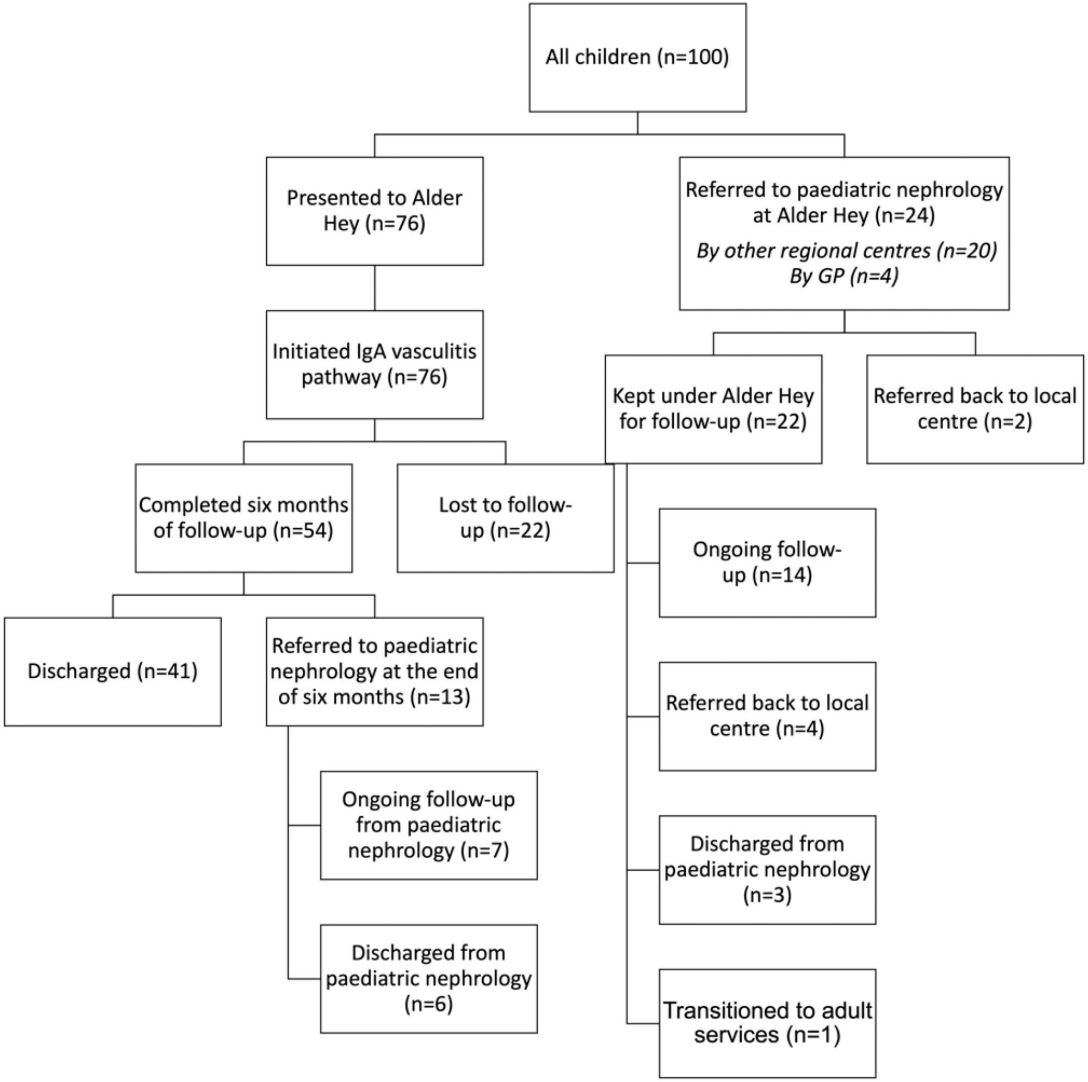
A summary of the pathways and routes of referral into Alder Hey Children’s Hospital. This comprises the outcomes, including discharge or ongoing follow-up, for the first 100 children recruited to the IgA Vasculitis Study. A total of 76% presented directly to our centre, while 4% presented originally to primary care and the rest of the children (20%) initially presented to general paediatric colleagues based at other hospitals in the region and were referred for secondary specialist care

The evolution of the IgA Vasculitis Study is summarized in Fig. 1. It commenced in June 2019 as a single-centre cross-sectional study that evolved into a longitudinal study in January 2021, incorporated wider tissue collection in February 2023 and received approval to become a trial-ready framework in October 2024. This trial-ready framework will optimize methods to capture patients from initial presentation with serial data and biosampling collection. To date, there have been 175 patients recruited to the study over the 5-year period (Figs. 2 and 3).

### Cohort characteristics

**Figure 4.**
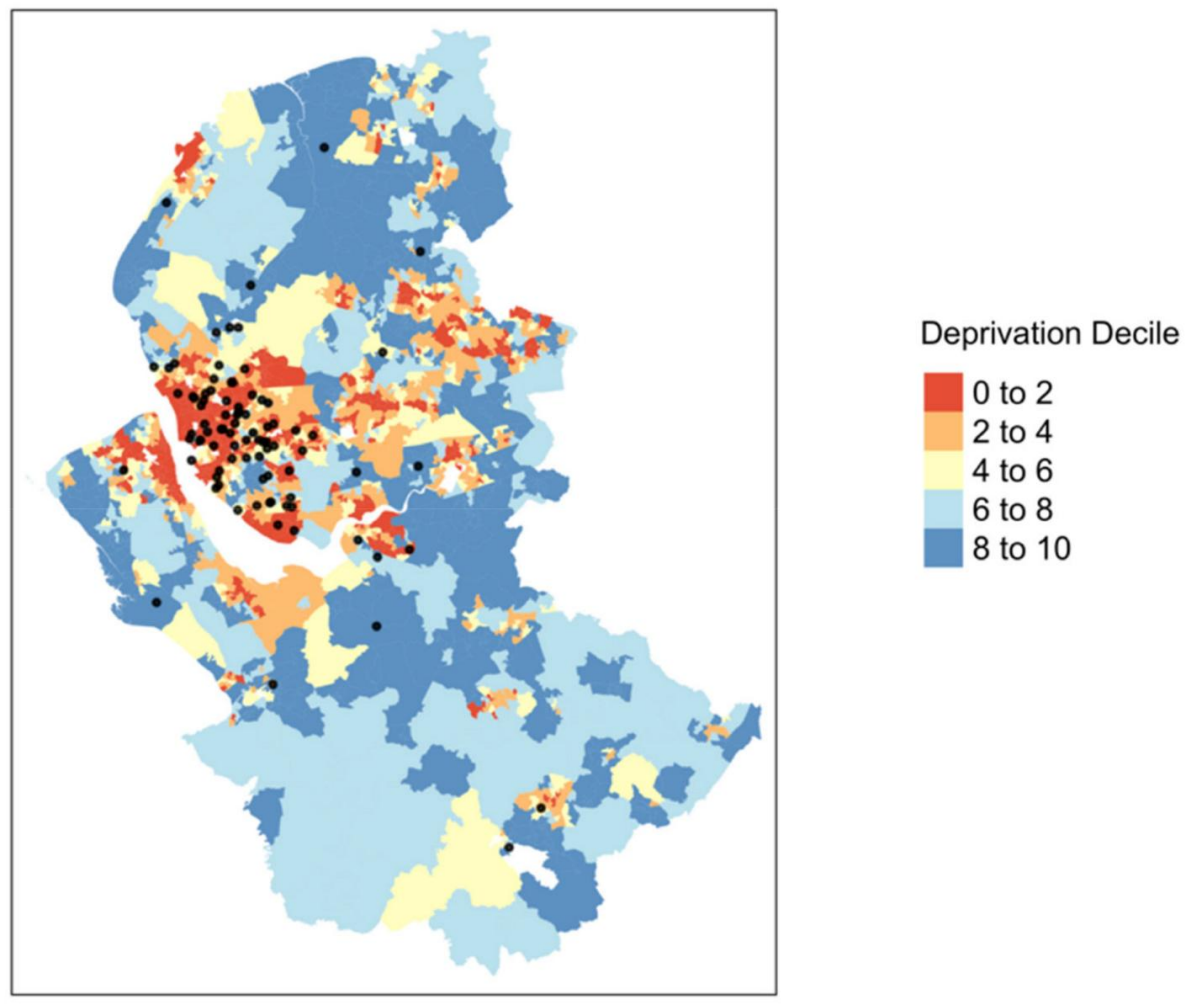
A heat map demonstrating geospatial distribution of participants in the IgA Vasculitis Study according to the IMD. To illustrate the socio-economic status of our cohort, the resident postcode for each patient was used to evaluate the IMD centile. The lowest IMD decile represents the greatest level of deprivation. Eight patients were excluded due to postcodes that could not be geocoded. (**a**) A view with the resident location of all participants and (**b**) a higher-resolution view focused on the Merseyside region

Data are presented for the first 100 recruits. Sixty children were male (1.5 male:1 female) and the mean age at diagnosis was 7.3 years (s.d. 3.7, range 1.0–17.0). Most children (85%) were Caucasian. Seasonal variation was observed, with peaks of incidence seen in the summer [*n* = 33 (33%)] and winter [*n* = 30 (30%)]. Fifty-six (56%) children lived in the lowest IMD quintile, with 38% living in the lowest decile (Fig. 4). The cohort was comparable to the local catchment population, where 63% reside in the lowest IMD quintile and 49% in the lowest decile [30].

Table 1 summarizes the presenting features of children in this cohort. All children presented with a lower limb–predominant rash. Seventy-six children reported either arthralgia or arthritis, with the ankle joints being the most frequently affected [*n* = 45 (59%)], followed by the knees [*n* = 27 (36%)]. GI involvement was reported in 43% of patients, mostly manifesting as abdominal pain [*n* = 38 (88%)], and one child presented with a GI haemorrhage. Multisystem involvement was present in 34 children. Preceding upper respiratory tract infections were reported in 57% of children, gastroenteritis was present in 3%, scarlet fever in 1% and bacterial tonsillitis in 1%. Other potential risk factors identified included recurrent bacterial tonsillitis (5%) and dental caries (2%). Atopy was documented in nine children (asthma, 5%; eczema, 3%; milk allergy, 1%).

**Table 1.** Summary of the clinical features at initial presentation for the children included in this cohort (*n* = 100).

Clinical features	Values
Rash (*n* = 100), *n* (%)	
Lower limb	100 (100)
Upper limb	26 (26)
Trunk	17 (17)
Face	5 (5)
Musculoskeletal involvement (*n* = 76), *n* (%)	
Oligoarticular	73 (96)
Polyarticular	3 (4)
Ankles	45 (59)
Knees	27 (36)
Wrists	14 (18)
Shoulders	1 (1)
Gastrointestinal involvement (*n* = 43), *n* (%)	
Pain	38 (88)
Vomiting	16 (37)
Diarrhoea	8 (19)
Gastrointestinal bleeding	1 (2)
Other system involvement	
Scrotal pain/swelling	2 (2)

The key clinical and laboratory results are summarized in Table 2. Two children (3%) had a UACR >30 mg/mm, meeting the definition of nephritis at presentation, and 11 (15%) were hypertensive. Of the children who did have blood tests performed, one (2%) child was anaemic and 26% had an elevated white cell count. No patients were thrombocytopaenic. Most children had normal serum albumin [38.5 g/l (s.d. 3.8), range 28–48]. The mean serum creatinine was also normal [mean 37.4 µmol/l (s.d. 11.3), range 20–88]. One child (2%) had an elevated serum creatinine of 88 µmol/l, corresponding to an estimated glomerular filtration rate of 46.5 ml/min/1.73 m^2^ at presentation. There were no available baseline data at presentation for 24 (24%) patients. The clinical course of the cohort is presented in Fig. 3.

**Table 2.** Key clinical and laboratory characteristics at presentation of children with a new diagnosis of IgAV (*n* = 76)

Characteristics at presentation	Values
Clinical and laboratory	
Blood pressure, *n* (%)	75 (99)
Systolic, mmHg, mean (s.d.) (range)	105.6 (12.0) (77–137)
Diastolic, mmHg, mean (s.d.) (range)	68.0 (12.9) (42–102)
Hypertensive, *n* (%)	11 (15)
Temperature, *n* (%)	76 (100)
Temperature, °C, mean (s.d.) (range)	37.0 ± 0.6 (36.0-39.5)
Febrile, *n* (%)	4 (5)
Urinalysis, *n* (%)	76 (100)
Normal urinalysis, *n* (%)	54 (71)
Isolated proteinuria, *n* (%)	16 (21)
Isolated haematuria, *n* (%)	3 (4)
Mixed proteinuria and haematuria, *n* (%)	3 (4)
UACR, *n* (%)	17 (22)
UACR, mg/mmol, mean (s.d.) (range)	32.7 (95.6) (0.5–398.0)
Blood results	
Full blood count, *n* (%)	55 (73)
Haemoglobin, g/l, mean (s.d.) (range)	123.6 (10.3) (98–157)
White cell count, ×10^9^/l, mean (s.d.) (range)	10.9 (4.5) (4.4–26.4)
Platelets, ×10^9^/l, mean (s.d.) (range)	331.5 (110.0) (160–660)
Neutrophils, ×10^9^/l, mean (s.d.) (range)	6.1 (3.9) (1.6–23.2)
Biochemistry	
Serum creatinine, µmol/l, mean (s.d.) (range)	37.4 (11.3) (20–88)
Serum albumin, g/l, mean (s.d.) (range)	38.5 (3.8) (28–48)

In total, 23% (23/100) of children met the definition of IgAV-N over the course of the study, with the mean age at disease presentation for this subgroup being 9.7 years (s.d. 3.1) compared with 6.6 years (s.d. 3.5) in the children with no nephritis (*P* < 0.001). Children with IgAV-N were more likely to reside in more affluent areas (mean IMD 5.4 *vs* 3.1, *P* = 0.003), although the number of children living in affluent regions was small. Most children who developed nephritis did so during the monitoring period (3%) or had established nephritis prior to specialist referral from another centre (18%). The mean time of nephritis onset was 25.5 days (s.d. 22.9) following disease presentation (range 0.0–101 days), excluding one child with an atypical, persisting disease course during which they had severe extrarenal manifestations for >1 year and developed nephritis 522 days after initial symptom development. Excluding this child, 15/22 (68%) children developed IgAV-N within 1 month following symptom onset, 20/22 (91%) children within 2 months, 21/22 (96%) within 3 months and all (100%) within 4 months.

The mean UACR for those with nephritis was 543.2 mg/mmol (s.d. 509.4, range 79.0–2357.7). One child developed nephrotic syndrome. According to the SHARE guideline definitions [17], 7% (7/100) met the definition of mild nephritis and did not require a kidney biopsy. The remaining children had moderate nephritis [16% (16/100)] and all had a kidney biopsy. Most children who had a biopsy [63% (10/16)] demonstrated ISKDC stage IIIb nephritis on the kidney histology, with histological stages II [6% (1/16)] and IIIa [13% (2/16)] seen in three patients. Two patients clinically evolved into a phenotype of IgA nephropathy and both biopsies were M1E1S0T0-C0 as per the Oxford MEST-C classification [31, 32]. One child had a kidney biopsy demonstrating C3 glomerulonephritis.

### Management of nephritis

Of the 23 children with nephritis, 6 (26%) required no treatment and remission was achieved spontaneously. Three children received renin–angiotensin–aldosterone system (RAAS) blockade as monotherapy and seven received a course of glucocorticoid therapy ± RAAS blockade. All other children (*n* = 7) received at least two immunosuppressants with varying combinations of oral (*n* = 7) or IV glucocorticoids (*n* = 3), azathioprine (AZA; *n* = 4), mycophenolate mofetil (MMF; *n* = 3) and cyclophosphamide (*n* = 1). The mean duration of follow-up for children with IgAV-N was 28.70 months (s.d. 21.97, range 2.40–79.90). At last review, 15 (65%) children were receiving no treatment, 4 (17%) were still receiving RAAS blockade and 4 (17%) remained on at least one immunosuppressant.

### Atypical disease

A recent report described atypical IgAV patients, some of whom were also participants of this current cohort [13]. In this current study, acknowledging that it is a specialist centre, eight children were diagnosed with atypical IgAV (recurrent, *n* = 3; persisting, *n* = 5), representing 8% of the total cohort. The male:female ratio was 1:1. Compared with the typical cases, these children were significantly older at diagnosis [11.1 years (s.d. 3.7) *vs* 7.0 years (s.d. 3.6), *P* = 0.006] and required longer follow-up [25.1 months (s.d. 55.0) *vs* 11.3 months (s.d. 14.5), *P* < .001]. Three had concomitant IgAV-N while the remaining children suffered from recurrent or persisting extrarenal manifestations. One child received no treatment, one was treated with dapsone, one with hydroxychloroquine, two with MMF and two with AZA. Four children received glucocorticoids and the three children with atypical IgAV and concomitant nephritis also received RAAS blockade.

### Clinical characteristics associated with the presence of nephritis and outcomes

Older age at onset [OR 1.24 (95% CI 1.09, 1.42)] and higher IMD [OR 1.27 (95% CI 1.09, 1.49)] were statistically significantly associated with the development of IgAV-N. GI involvement and positive urine dipstick (for proteinuria and/or haematuria) at baseline were also strongly associated with the risk of developing nephritis [OR 3.43 (95% CI 1.16, 10.45) and OR 20.03 (95% CI 2.66, 483.6), respectively]. A negative urine dipstick at baseline had a positive predictive value of 98.2% for not developing IgAV-N. Other parameters including ethnicity, sex, season of onset, preceding viral illness, extensive rash, joint involvement and multisystem involvement were not associated with nephritis. No association was found between abnormal blood tests at baseline (leucocytosis, neutrophilia, thrombocytosis, elevated CRP, hypoalbuminaemia) and nephritis. Following initial presentation, most children (*n* = 75) had no further A&E attendances, while 11% had a second A&E attendance related to symptoms of IgAV. One child diagnosed with persisting IgAV attended A&E on 10 occasions due to arthralgia, arthritis and abdominal pain. There was a trend towards a lower mean number of A&E presentations in typical cases compared with atypical cases [0.4 (s.d. 0.9) *vs* 1.9 (s.d. 3.5), respectively; *P* = 0.162] and a similar trend was seen regarding unplanned hospitalizations [0.3 (s.d. 0.5) *vs* 0.5 (s.d. 0.5), respectively; *P* = 0.113]. Patients with nephritis had a greater mean number of A&E presentations when compared with those without nephritis, but this was not statistically significant (0.8 *vs* 0.4; *P* = 0.083). Children with nephritis did have significantly more hospital admissions than those without nephritis [mean 0.7 (s.d. 0.7) *vs* 0.2 (s.d. 0.4), respectively; *P* < 0.001] and children with nephritis (*n* = 23) *vs* those without (*n* = 77) had a greater mean number of hospital appointments [6.2 (s.d. 5.9) *vs* 13.4 (s.d. 8.1), respectively; *P* < 0.001]. The overall rate of did-not-attend appointments was 8%, and this did not vary significantly according to the presence of nephritis (7.9% *vs* 3.2%, respectively; *P* = 0.241). At the end of the study follow-up period, 50% of children had been discharged, 22% were lost to follow-up and presumed to have a normal outcome, 21% were undergoing active follow-up by a paediatric nephrologist, 6% had been referred to their local centre and one (1%) child was transitioned to adult care (Fig. 3). No children required kidney replacement therapy.

## Discussion

This study presents the evolution of a cohort study aimed at advancing the field for IgAV as a foundation for future evidence generation using a trial-ready framework. The evolutionary stages of the study have generated data on the natural history of the disease that continues to highlight the burden of nephritis and a description of the first 100 children recruited allows meaningful comparison with previously published cohorts. To our knowledge, this is the largest prospective longitudinal cohort study dedicated to recruiting children with IgAV with a corresponding invaluable bioresource [11, 19]. The descriptive cohort also highlights the subgroup of children who experience an atypical disease course that may have similarities to adult-onset disease. Consistent with the literature, we have highlighted several risk factors for the development of IgAV-N, including older age of disease onset, GI involvement and a positive urinalysis (for proteinuria and/or haematuria) during the early phase of the disease. Additionally, we have highlighted many referrals for specialist input, a high lost-to-follow-up rate during the monitoring phase and hospital re-attendances and admissions, which were in greater demand in patients with nephritis. These are likely to reflect underrecognized costs in the wider consequences of nephritis associated with this condition.

The cohort is representative because the demographics align with the current literature, demonstrating a slight male predominance, high proportion of Caucasian children and the majority of cases diagnosed at <10 years of age [4]. This study identified a 23% rate of nephritis, slightly lower than previous reports in the literature of 30–60%, which may reflect the heterogeneity in the definitions of nephritis used within the literature [33, 34]. The strength of proteinuria and its relationship with disease outcome continues to support its use as an early marker to risk stratify patients, and as an outcome measure for clinical trials, aligned with other forms of glomerulonephritis. The primary outcome measures of previous trials in IgAV, although few, typically span durations of 12–30 months and have focused on resolution of proteinuria as the study endpoint, with secondary outcome measures including the need for additional treatment, resolution of haematuria, vasculitis disease activity and preserving normal kidney function [35].

We identified a low rate of recurrent disease (3%), although reported recurrence rates in the literature vary widely, ranging from 2.6 to 66%, and this is likely due to the lack of standardized definitions and disease heterogeneity [13]. In two large recent retrospective cohorts in the literature by Ekinci *et al.* [36] and Karadag *et al.* [37], disease recurred in 16.4% and 4.6%, respectively. Recurrent and/or persisting disease is a significant burden for patients and families, and there is very little evidence to guide the management of these patients [13]. Consideration of patients experiencing an atypical disease course within a clinical trial setting is vital and must include consensus and standardized definitions of recurrence, particularly with key discriminators indicating when rescue treatment may be required. Similarly, patients with atypical extrarenal manifestations should be explicitly included in trials primarily designed to evaluate novel treatments for nephritis.

The focus of this descriptive cohort was to identify the early evolution of nephritis to direct opportunities for intervention. The rates of glucocorticoid use in IgAV-N vary significantly in the literature, from 36 to 89% in children, and similar rates of 37–86% in adults [5, 15, 38–41]. Our cohort demonstrated a slightly lower rate of glucocorticoid use (30%), which may be in keeping with increasing awareness of steroid-related toxicity and the growing reluctance to use these agents in the absence of evidence. Additionally, we identified that 26% of children with nephritis were conservatively managed, comparable to the findings by Selewski *et al.* [41], who reported conservative management for 24% of paediatric patients and 12% of adults. The conservative approach and high rate of resolution of nephritis emphasizes the need for carefully designed trials, with accurate ways to truly identify patients at risk of poorer outcomes [42].

This work is leading to the design of age-inclusive clinical trials for IgAV-N with proposed trial interventions during the first 12 months, early identification of higher risk individuals and acknowledging the differences in short- and long-term kidney outcomes seen across different ages.

There are limitations relevant to this study, as it presents a relatively small and heterogeneous cohort with missing data, particularly related to children who did not complete the full duration of kidney monitoring and in those who initially presented to other centres. Additionally, the setting was a tertiary referral centre for paediatric nephrology with expertise in IgAV, thus it is likely to encounter more patients with IgAV-N and atypical disease. There are also significant confounders, particularly related to the COVID-19 pandemic regarding disruption of study recruitment, adherence to the monitoring pathway and follow-up appointments.

## Conclusion

This report describes a UK-based study dedicated to advancing paediatric IgAV that has the potential to act as a global exemplar and continues to highlight the unmet needs of patients with IgAV that is mostly related to nephritis. A global effort to conduct multicentre clinical trials is urgently needed to alter the disease course for children with IgAV.

## Data Availability

All data are presented in the article. Any raw data will be made available upon reasonable request to the corresponding author.
